# Brilacidin Demonstrates Inhibition of SARS-CoV-2 in Cell Culture

**DOI:** 10.3390/v13020271

**Published:** 2021-02-09

**Authors:** Allison Bakovic, Kenneth Risner, Nishank Bhalla, Farhang Alem, Theresa L. Chang, Warren K. Weston, Jane A. Harness, Aarthi Narayanan

**Affiliations:** 1 National Center for Biodefense and Infectious Diseases, George Mason University, Manassas, VA 20110, USA; abakovic@masonlive.gmu.edu (A.B.); krisner@masonlive.gmu.edu (K.R.); nbhalla@gmu.edu (N.B.); falem@gmu.edu (F.A.); 2 Public Health Research Institute, Rutgers, New Jersey Medical School, The State University of New Jersey, Newark, NJ 07103, USA; changth@njms.rutgers.edu; 3 Innovation Pharmaceuticals Inc., Wakefield, MA 01880, USA; kyle@ipharminc.com (W.K.W.); jharness@ipharminc.com (J.A.H.)

**Keywords:** brilacidin, coronavirus, antiviral, defensin, peptidomimetic, entry inhibition

## Abstract

Severe Acute Respiratory Syndrome Coronavirus 2 (SARS-CoV-2), the newly emergent causative agent of coronavirus disease-19 (COVID-19), has resulted in more than two million deaths worldwide since it was first detected in 2019. There is a critical global need for therapeutic intervention strategies that can be deployed to safely treat COVID-19 disease and reduce associated morbidity and mortality. Increasing evidence shows that both natural and synthetic antimicrobial peptides (AMPs), also referred to as Host Defense Proteins/Peptides (HDPs), can inhibit SARS-CoV-2, paving the way for the potential clinical use of these molecules as therapeutic options. In this manuscript, we describe the potent antiviral activity exerted by brilacidin—a de novo designed synthetic small molecule that captures the biological properties of HDPs—on SARS-CoV-2 in a human lung cell line (Calu-3) and a monkey cell line (Vero). These data suggest that SARS-CoV-2 inhibition in these cell culture models is likely to be a result of the impact of brilacidin on viral entry and its disruption of viral integrity. Brilacidin demonstrated synergistic antiviral activity when combined with remdesivir. Collectively, our data demonstrate that brilacidin exerts potent inhibition of SARS-CoV-2 against different strains of the virus in cell culture.

## 1. Introduction

The global coronavirus disease-19 (COVID-19) pandemic resulting from infection by Severe Acute Respiratory Syndrome Coronavirus 2 (SARS-CoV-2), the novel coronavirus, has resulted in excess of 100 million infections and 2.1 million deaths worldwide, including over 25 million cases and 425,000 fatalities in the United States [1].

Approximately 15% of COVID-19 patients will develop lung injury, including severe respiratory distress that can progress to Acute Respiratory Distress Syndrome (ARDS), often requiring prolonged ventilator support and leading to death. Intensive care units, hospitals, and health care systems risk becoming overwhelmed by critically ill COVID-19 patients. COVID-19 itself is characterized by a heightened inflammatory component, with several proinflammatory cytokines, such as TNF-α, IL-1β, and IL-6, strongly upregulated in infected individuals [2,3,4]. The prevalence of secondary bacterial infections [5,6,7,8], which can occur in up to 20% of cases among hospitalized patients, reinforces the need for a multipronged treatment approach that can address the complexities of COVID-19.

To date, only two vaccines have received Emergency Use Authorization from the FDA to prevent SARS-CoV-2 infection, alongside few modestly effective therapies to treat COVID-19, with remdesivir the only FDA-approved treatment [9]. Therapies are predominately being developed to treat acute viral infections in hospital settings versus being evaluated for their prophylactic potential [10,11,12]. It is likely multiple interventional strategies will be required to prevent and treat COVID-19 [13,14], particularly given the emergence of drug- and vaccine-resistant variants, with SARS-CoV-2 possibly becoming an endemic viral infection [15]. Drugs shown to exhibit broad spectrum antiviral activity might help address COVID-19 and potential future viral pandemics [16].

Natural and synthetic antimicrobial peptides (AMPs), also called Host Defense Proteins/Peptides (HDPs), comprise potentially effective countermeasures against COVID-19, having shown inhibitory activity against multiple viruses [17,18,19,20,21,22,23,24,25,26]. Integral components of the innate immune response, HDPs are typically small (12–80 amino acids) proteins and peptides expressed widely in the animal kingdom that serve as the “first line of defense” against foreign pathogens and potential subsequent infection and related inflammation. HDPs have been recognized as potential sources for promising therapeutics [27,28,29,30,31,32,33,34,35]. In mammals, HDPs are found within granules of neutrophils and in secretions from epithelial cells covering skin and mucosal surfaces.

Despite a variety of chemical sequences, as well as secondary and tertiary structures, most HDPs share an amphiphilic topology—with a positively charged face on one side and a hydrophobic face on the other side [36]. In contrast, most bacteria express a negative charge on their outer membranes [37], as well as lack cholesterol, an essential component of mammalian membranes [38,39]. Due to these differences, HDPs can selectively and variously interact with pathogens, increasing their susceptibility to proteolysis and degradation [40].

SARS-CoV-2 infection has been shown to suppress defensins, a type of HDP, suggesting that the innate immunity provided by defensins may be compromised [41]. Suppression of LL37, a human cathelicidin (another type of HDP) shown to possess immunomodulatory and antiviral properties [42], has also been observed in COVID-19 patients [43]. Increased expression of defensins and cathelicidins (e.g., LL37) can decrease both the viral and inflammatory load in the context of several respiratory viral infections [44], further supporting the protective role of HDPs. Numerous recently published reports [45,46,47,48,49,50,51,52] support the antiviral activity of HDPs in the context of coronaviruses, including SARS-CoV-2.

**Figure 1:**
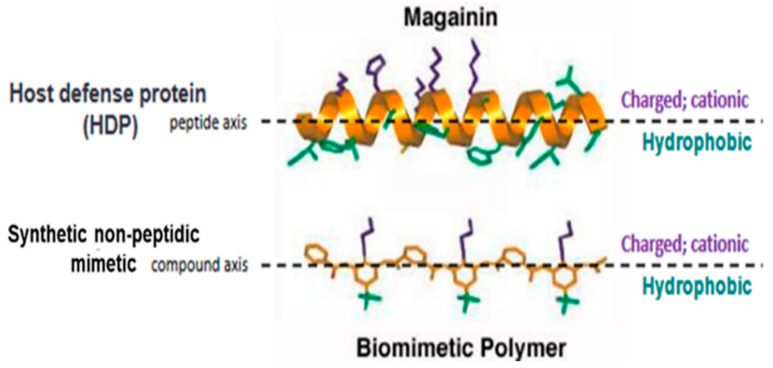
Structure of brilacidin. Schematic representation of the amphiphilic α-helix structure of the Host Defense Protein/Peptide (HDP) magainin above that of a synthetic nonpeptidic mimetic polymer, such as brilacidin, capturing the structural and biological properties of HDPs using fully synthetic, nonpeptidic scaffolds and sidechains.

Brilacidin (PMX-30063) is a synthetic, nonpeptidic, small-molecule mimetic of HDPs [53,54,55,56,57,58] (Figure 1). Building on “first principles” in medicinal chemistry, rational design tools—leveraging sophisticated informatics to fine-tune physicochemical properties and structure–activity relationships [59,60,61]—enabled brilacidin (an arylamide foldamer [62,63]) to overcome the shortcomings and challenges that have complicated the clinical development of natural HDPs [64]. These include proteolytic degradation, toxicity, lack of efficacy, malabsorption, and high cost to produce. In contrast to natural HDPs, as well as other HDP analogs, brilacidin was designed de novo [65,66,67] to be much smaller, more stable, more potent, more selective, and more economical to manufacture. Brilacidin has already been successfully tested in clinical trials and shown to exhibit potent antibacterial activity in Phase 2 clinical trials for treatment of Acute Bacterial Skin and Skin Structure Infections (ABSSSI), and anti-inflammatory activity, as supported in Phase 2 clinical trials for treatment of Ulcerative Proctitis/Ulcerative Proctosigmoiditis. As such, brilacidin has already demonstrated HDP-like therapeutic properties in the clinic.

Within the broader context of the global COVID-19 pandemic and the potential therapeutic role for HDPs, brilacidin was evaluated to determine if the drug might exhibit antiviral properties against SARS-CoV-2.

## 2. Materials and Methods

### 2.1. Cell Culture

Vero African green monkey kidney cells (ATCC, CCL-81) and Calu-3 human lung epithelial cells (ATCC, HTB-55) were obtained from the American Type Culture Collection. Vero cells were cultured in Dulbecco’s Modified Eagle’s Medium (DMEM, Quality Biological, 112-013-101CS, Gaithersburg, MD, USA) supplemented with 4.5 g/L glucose, 2 mM L-glutamine (FisherSci, MT2005CI, Chicago, IL, USA), 5% heat-inactivated fetal bovine essence (FBE) (VWR, 10805-184) for Vero cells, 10 μg/mL streptomycin, and 10 U/mL penicillin (VWR, 45000-652). Calu-3 cells were cultured in Eagle’s Minimum Essential Medium (EMEM, VWR, 670086) supplemented with 10% fetal bovine serum (FBS) (ThermoFisher, 10437028, Carlsbad, CA, USA). All cell lines were cultivated at 37 °C and 5% CO_2_.

### 2.2. Inhibitors

Brilacidin (as brilacidin tetrahydrochloride) was provided by Innovation Pharmaceuticals Inc. (Wakefield, MA, USA) and dissolved in dimethyl sulfoxide (DMSO) (Fisher Scientific, BP231). Hydroxychloroquine sulfate (SelleckChem, S4430, Houston, TX, USA), remdesivir (MedChemExpress, HY-104077, Monmouth Junction, NJ, USA), and favipiravir (FisherScientific, NC1312443) were obtained and dissolved in DMSO.

### 2.3. Toxicity Screens

Cells were seeded in 96-well white plates 24 h prior as follows: Vero and Caco-2 cells at 5 × 10^4^ cells per well, Calu-3 cells at 1.3 × 10^5^ cells per well. Inhibitors were diluted to the desired micromolar concentration (μM) in the appropriate culture media. Diluted compounds were added to the cells and plates were incubated at 37 °C, 5% CO_2_. At 24 h post-treatment (hpt), culture media were removed from the cells and cell viability was measured using a CellTiter-Glo^®^ Luminescent Cell Viability Assay per manufacturer’s instructions (Promega, G7572, Madison, WI, USA). Luminescence was measured using a Beckman Coulter DTX 880 Multimode plate reader with Multimode Analysis Software Version 3.3.0.9.

### 2.4. SARS-CoV-2 Infections

SARS-CoV-2 (Washington strain 2019-nCoV/USA-WA1/2020) was obtained from BEI Resources (NR-52281) and was used for all infections, unless otherwise specified. For all infections, cells were seeded in 96-well plates 24 h prior as follows: Vero cells at 5 × 10^4^ cells per well, Calu-3 cells at 1.3 × 10^5^ cells per well. Inhibitors were dissolved in DMSO and diluted in culture media to the indicated concentrations such that the final concentration of DMSO in the treatment was ≤0.1%. Mock-infected cells were included as untreated and uninfected controls during all infections. Cells were pretreated with media containing drug or 0.1% DMSO vehicle control for 2 h prior to infection. For nondirect viral infections, virus was diluted in culture media to the indicated multiplicity of infection (MOI) and this inoculum was overlaid on cells for 1 h. For direct viral infections, virus was diluted to the indicated MOI in culture media containing 0.1% DMSO or the inhibitor at the indicated concentration, and this virus:inhibitor solution (i.e., treated inoculum) was incubated at 37 °C and 5% CO_2_ for 1 h. After this incubation, the treated inoculum was overlaid on cells for 1 h. Conditioned media containing inhibitor or standard media were added to cells after removal of virus. For synergy experiments, fresh media containing inhibitor(s) were added to cells after removal of virus. Plates were incubated at 37 °C, 5% CO_2_ for the indicated duration. At the indicated hour time point postinfection (hpi), viral supernatants were collected and stored at −80 °C or used immediately for assays.

### 2.5. Plaque Assay

Vero cells were plated in 12-well plates at a density of 2 × 10^5^ per well and incubated for 24 h. Infection supernatants were serially diluted to 10-4 in culture media and overlaid on cells for 1 h. Cells were covered with Eagle’s Minimum Essential Medium (without phenol red, supplemented with 5% FBE, nonessential amino acids, 1 mM sodium pyruvate (VWR, 45000-710, Dixon, CA, USA), 2 mM L-glutamine, 20 U/mL penicillin, and 20 μg/mL streptomycin) with 0.6% agarose (ThermoFisher, 16500100). At 48 hpi, cells were fixed with 10% formaldehyde (FisherSci, F79P-4) for 1 h. Medium was removed, wells were washed with diH_2_O and stained with a 1% crystal violet (FisherSci, C581-25) and 20% ethanol solution (FisherSci, BP2818-4). Plaque assay datasets are represented as both plaque forming units per milliliter (PFU/mL) and as percentage of virus titer versus the DMSO control.

### 2.6. RNA Extraction and RT-PCR

At the indicated time points postinfection, cells were washed with 1X PBS and lysed with TRIzol Reagent (Invitrogen). Intracellular RNA was extracted using Direct-zol Miniprep RNA kit (Zymo Research, R2051s) per manufacturer’s instructions. Extracted viral RNA was stored at −80 °C or used immediately for analysis by RT-PCR. Primers and probes for detection of SARS-CoV-2 RNA specific to ORF 1ab for the envelope (E) and nucleocapsid (N) viral genome fragments were obtained from Integrated DNA Technologies (10006821, 10006822, 10006823, Coralville, IA, USA). The probe was double-quenched with ZEN/IBFQ and contained a 6-FAM fluorescent dye attachment at the 5′ end. 18S rRNA endogenous control primer/probe set was utilized for semiquantitative RT-PCR normalization (ThermoFisher, 4333760T). Thermal cycling conditions were adapted from Verso 1-step RT-qPCR kit (ThermoFisher, AB4101C) per the manufacturer’s instructions: 1 cycle at 50 °C for 20 min, 1 cycle at 95 °C for 15 min, 40 cycles at 95 °C for 15 s with 52 °C (CoV2 E,N) and 60 °C (18S) for 1 min using StepOnePlus™ Real-Time PCR System with StepOne™ Software Version 2.3 (Carlsbad, CA, USA). No template controls and mock infections were included for all analyses and established the limits of detection. Quantitative values were calculating using the ΔΔCt method [68] with viral entities normalized to 18S levels and fold-changes calculated versus mock-infected conditions. Datasets are represented as both raw values calculated for fold-change and as a percentage versus the DMSO condition.

### 2.7. Statistical Analyses

Graphs represent the mean ± SD for all data obtained, with the exception of Figure 4A,B, for which sigmoidal Hill-type models as a function of brilacidin tetrahydrochloride concentration were fit to the data (using nonlinear least-squares regression in NONMEM Version 7.4, as performed by Enhanced Pharmacodynamics (ePD) on behalf of Innovation Pharmaceuticals Inc.). Statistical analyses and significance for all other figures were determined using One-Way ANOVA with Dunnett’s Post Test in Prism 7 (Graph Pad) unless otherwise stated. Significance values are indicated using asterisks for * *p* < 0.0332, ** *p* < 0.0021, *** *p* < 0.0002, **** *p* < 0.0001, ns for not significant.

## 3. Results

### 3.1. Brilacidin Inhibits SARS-CoV-2 Replication in Vero Cells

**Figure 2:**
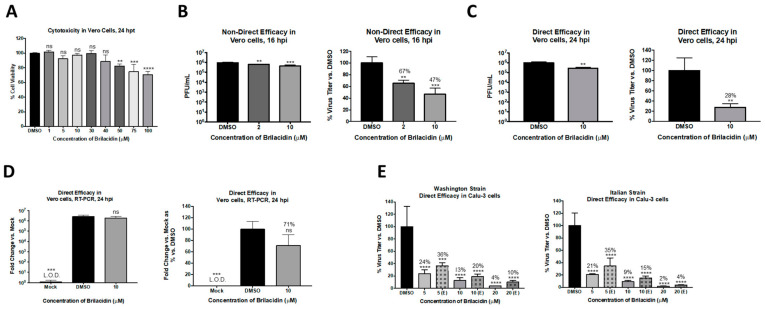
Brilacidin displays statistically significant inhibitory efficacy against Severe Acute Respiratory Syndrome Coronavirus 2 (SARS-CoV-2) in two cell types and against two different strains. (**A**) Vero cells were treated at various concentrations of brilacidin, and cell viability was measured versus the dimethyl sulfoxide (DMSO) control at 24 h post-treatment (hpt) as described in Materials and Methods. (**B**) Vero cells were pretreated for 2 h with 2 or 10 μM brilacidin, infected with SARS-CoV-2 nondirectly at multiplicity of infection (MOI) 0.1 for 1 h, and post-treated with media containing brilacidin as described in Materials and Methods. At 16 h postinfection (hpi), viral supernatants were evaluated by plaque assay as described in Materials and Methods. Vero cells were pretreated for 2 h with 10 μM brilacidin. SARS-CoV-2 was diluted to MOI 0.1 in culture media containing brilacidin and incubated for 1 h. Viral inoculum containing inhibitor was added to cells for 1 h for a direct infection and post-treated with media containing brilacidin as described in Materials and Methods. At 24 hpi, (**C)** viral supernatants were evaluated by plaque assay, and (**D**) total RNA was extracted from intracellular lysates and viral RNA was quantified by RT-PCR as described in Materials and Methods. (**E**) Calu-3 cells were pretreated for 2 h with varying concentrations of brilacidin (5, 10, or 20 μM) or media only (indicated as [E, entry]). SARS-CoV-2 was diluted to MOI 0.1 in culture media containing brilacidin (5, 10, or 20 μM) and incubated for 1 h. Brilacidin-treated viral inoculum was added to cells for 1 h for a direct infection and replaced with inhibitor containing media or media only (indicated as [E, entry]) as described in Materials and Methods. At 24 hpi, viral supernatants were evaluated by plaque assay as described in Materials and Methods. Graphs are representative of one independent experiment performed in technical triplicates (*n* = 3). ** *p* < 0.0021, *** *p* < 0.0002, **** *p* < 0.0001.

As a first step, the potential of brilacidin to exert an antiviral activity against SARS-CoV-2 was assessed using Vero cells as an infection model. Toxicity assessment of brilacidin in Vero cells was initially performed by incubating the cells with increasing concentrations of the compound for 24 h, after which cell viability was assessed by Cell Titer Glo assay (Figure 2A). Brilacidin, at up to 40 µM concentration, did not affect cell viability when compared to the DMSO vehicle control; a dose-dependent, statistically significant decrease in cell viability was detected at higher concentrations. The effect of brilacidin treatment on SARS-CoV-2 viral replication was then evaluated in Vero cells by plaque assay. Vero cells were pretreated with brilacidin for 2 h, after which media containing the drug were removed and replaced with virus inoculum (Washington strain 2019-nCoV/USA-WA1/2020). Infection was allowed to progress for 1 h, after which the inoculum was removed and replaced with brilacidin containing media. Cell culture supernatants from vehicle-treated and brilacidin-treated cells were collected at 16 h postinfection, and the SARS-CoV-2 infectious titer in the supernatants was quantitated by plaque assay and compared to the DMSO-treated control. The data demonstrate that brilacidin treatment resulted in a dose-dependent decrease in infectious viral titer with a maximum of 53% inhibition of virus observed in the presence of the higher concentration of the compound (10 µM) that was tested (Figure 2B).

### 3.2. Brilacidin Appears to Disrupt the Integrity of the SARS-CoV-2 Virion in a Manner That Interferes with Entry

The potential for brilacidin to interfere directly with the virus prior to cell attachment was assessed. The hypothesis was that if brilacidin is able to impact viral integrity, inhibition of SARS-CoV-2 (Washington strain 2019-nCoV/USA-WA1/2020) should increase above that observed in the assay (Figure 2C) when modified to include a brilacidin-treated inoculum. To evaluate this, the inoculum was independently incubated with 10 µM brilacidin for 1 h, after which the treated inoculum (virus + brilacidin) was used to infect Vero cells. The infection was, thus, also carried out in the presence of brilacidin for 1 h, after which the inoculum was removed and replaced with media containing the drug. The culture supernatants were assessed for viral load by plaque assay at 24 h postinfection. The outcomes of this experiment revealed a higher inhibition of SARS-CoV-2 (72% inhibition), alluding to an inhibitory activity exerted upon the virus directly (Figure 2C). Using the same assay (for 10 µM brilacidin), the intracellular viral genomic copy numbers were assessed by semiquantitative RT-PCR at 24 h postinfection (Figure 2D), which demonstrated a 29% decrease in the viral genomic copies with brilacidin treatment; this extent of inhibition of intracellular RNA copies assessed at later timepoints in infection is not unexpected for an inhibitor with likely activity exerted during the early entry and postentry steps.

To independently assess the impact of brilacidin on the virion and thus add support to the role of brilacidin as a potential inhibitor of viral entry, a virus inhibition assay was conducted akin to virus neutralization observed in the presence of antibodies. This assay was performed using two different strains of SARS-CoV-2 (Washington strain-nCoV/USA-WA1/2020 and Italy strain-Italy-INMI1) in Calu-3 cells, a human lung cell line. To that end, SARS-CoV-2 inoculum was incubated with brilacidin at varying concentrations (5, 10, or 20 µM) for 1 h, after which the treated inoculum was used to infect Calu-3 cells. In this experiment, the cells were not pretreated with the inhibitor (brilacidin) prior to the infection nor post-treated (referenced as (E) in the Figure 2E). Standard pre- and post-treatment conditions were run alongside as controls to compare the impact of brilacidin treatment on the virus alone. The infectious virus titer in the supernatant was quantified by plaque assay, which revealed marked reductions of virus titer (Figure 2E, indicated as [E, entry]). The level of inhibition observed at entry was only slightly lower than that observed when the cells were pre- and post-treated with brilacidin concomitantly (Figure 2E), supporting the concept that brilacidin has an inhibitory effect on the virus in a manner similar to the neutralization of antibodies, potentially by disrupting viral integrity and thus impairing the virion’s ability to complete the viral entry process.

### 3.3. Brilacidin Inhibits SARS-CoV-2 in Calu-3 Cells

**Figure 3:**
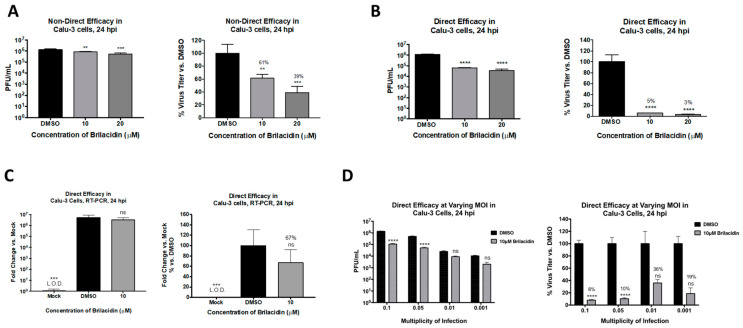
Brilacidin exhibits potent inhibition of SARS-CoV-2 in an ACE2-positive human lung cell line (Calu-3 cells). (**A**) Calu-3 cells were pretreated for 2 h with 10 or 20 μM brilacidin and infected with SARS-CoV-2 at MOI 0.1 nondirectly or (**B**,**C**) directly with brilacidin for 1 h as described in Materials and Methods. Cells were post-treated with media containing brilacidin, and at 24 hpi, viral supernatants were evaluated by plaque assay (**A**,**B**) or intracellular RNA extracted and viral RNA quantified by RT-PCR (**C**) as described in Materials and Methods. (**D**) Calu-3 cells were pretreated for 2 h with 10 μM of brilacidin, infected with SARS-CoV-2 at MOIs of 0.1, 0.05, 0.01, or 0.001 directly with brilacidin at 10 μM for 1 h, and post-treated with media containing brilacidin as described in Materials and Methods. At 24 hpi, viral supernatants were evaluated by plaque assay as described in Materials and Methods. Statistical analyses for varying MOIs was determined using Two-Way ANOVA with Sidak’s multiple comparisons test. Significance for all other graphs were determined as described in Materials and Methods. Graphs are representative of one independent experiment performed in technical triplicates (*n* = 3). ** *p* < 0.0021, *** *p* < 0.0002, **** *p* < 0.0001, ns = not significant.

To further ascertain that brilacidin can elicit anti-SARS-CoV-2 activity in an ACE2-positive human lung cell, additional experiments were conducted in the Calu-3 infection model. The toxicity of brilacidin in this cell line was initially assessed at 10 and 20 µM concentrations by incubating the cells with the compound for 24 h. The assay revealed that these concentrations of brilacidin were nontoxic to Calu-3 cells. The inhibitory effect of brilacidin in the Calu-3 cell line was first confirmed by pretreatment (for 2 h) and postinfection treatment (for 24 h) of cells with brilacidin, which demonstrated a dose-dependent decrease of viral load, with the higher concentration of brilacidin providing 61% inhibition of infectious viral titer (Figure 3A). However, when the experiment was modified to include a brilacidin-treated inoculum—with preincubation of the virus with brilacidin for 1 h prior to infection, and with infection carried out in the presence of the compound—the extent of inhibition dramatically increased, resulting in 95% and 97% reduction of infectious viral titer at the 10 and 20 µM concentration of the compound, respectively (Figure 3B). Quantification of intracellular viral RNA by semiquantitative RT-PCR at 24 h postinfection (for 10 µM brilacidin) demonstrated a 33% decrease in the viral genomic copies upon brilacidin treatment.

The inhibition of infectious virus titer as a variable of viral load was assessed by quantifying inhibition at lower multiplicities of infection (MOIs) with brilacidin at a fixed concentration of 10 µM. Interestingly, the inhibitory potential of brilacidin was best observed at the highest MOIs tested, with inhibition of virus at the lower MOIs (0.01 and 0.001) not showing statistical significance (Figure 3C). The inhibition exerted at the MOIs of 0.1 and 0.05 were comparable to each other.

### 3.4. Selectivity Index Determination for Brilacidin against SARS-CoV-2 in Calu-3 Cells

**Figure 4:**
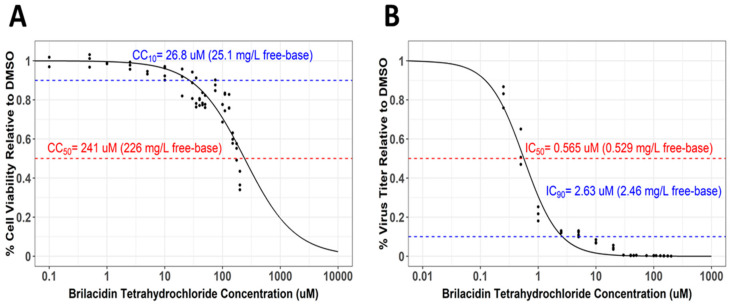
Brilacidin exhibits potent inhibition of SARS-CoV-2 in Calu-3 cells. (**A**) Calu-3 cells were treated at increasing concentrations of brilacidin at a range of 0.1–200 μM. Cell viability was measured at 24 hpt and calculated versus the DMSO control as described in Materials and Methods. (**B**) Calu-3 cells were pretreated for 2 h with brilacidin at increasing concentrations, directly infected with treated (and preincubated) viral inoculum at MOI 0.1 at the indicated pretreatment brilacidin concentration for 1 h, and post-treated with media containing brilacidin as described in Materials and Methods. At 24 hpi, viral supernatants were evaluated by plaque assay as described in Materials and Methods. Graphs are representative of one independent experiment performed in technical triplicates (*n* = 3). Sigmoidal Hill-type models as a function of brilacidin tetrahydrochloride concentration and were fit to the cell viability (**A**) and inhibitory response (**B**) data using nonlinear least-squares regression. The dashed lines indicate (**A**) derived cytotoxicity concentration (CC)10 and CC50 values, and (**B**) derived inhibitory concentration (IC)50 and IC90 values. The calculated Selectivity Index is 426 (CC50 = 241 μM/IC50 = 0.565 μM).

The Selectivity Index, a ratio that compares a drug’s cytotoxicity and antiviral activity, is a measure of how likely a drug is to be safe and effective when translated to human testing in the clinic. The 50% cytotoxicity concentration (CC50), that is, the concentration that results in the reduction of cell viability by 50%, is compared to the concentration that results in 50% of the maximal inhibitory response (IC50). The values for 90% cell viability (CC10) and the 90% inhibitory concentration (IC90) were also derived. The CC50 values for brilacidin in the context of Calu-3 cells were assessed by measuring cell survival over a concentration range between 0.1 and 200 µM, which revealed that the 50% reduction in cell viability was observed at a concentration of 241 μM, with 90% viability (CC10) observed at 26.8 μM, thus suggesting brilacidin was extremely well tolerated (Figure 4A). Quantification of the inhibitory response—when the virus was directly preincubated with brilacidin prior to infection; cells were treated prior to infection; brilacidin was present during infection; and infected cells were maintained in the presence of brilacidin postinfection (assay as in Figure 3B)— demonstrated that brilacidin achieved 90% inhibition at a concentration of 2.63 μM and 50% inhibition at 0.565 μM, yielding a Selectivity Index of 426 (CC50 = 241 μM/IC50 = 0.565 μM). (Figure 4B).

### 3.5. Brilacidin in Combination with Other Antiviral Treatments: Synergistic Activity against SARS-CoV-2 in Combination with Remdesivir in Calu-3 Cells

**Figure 5:**
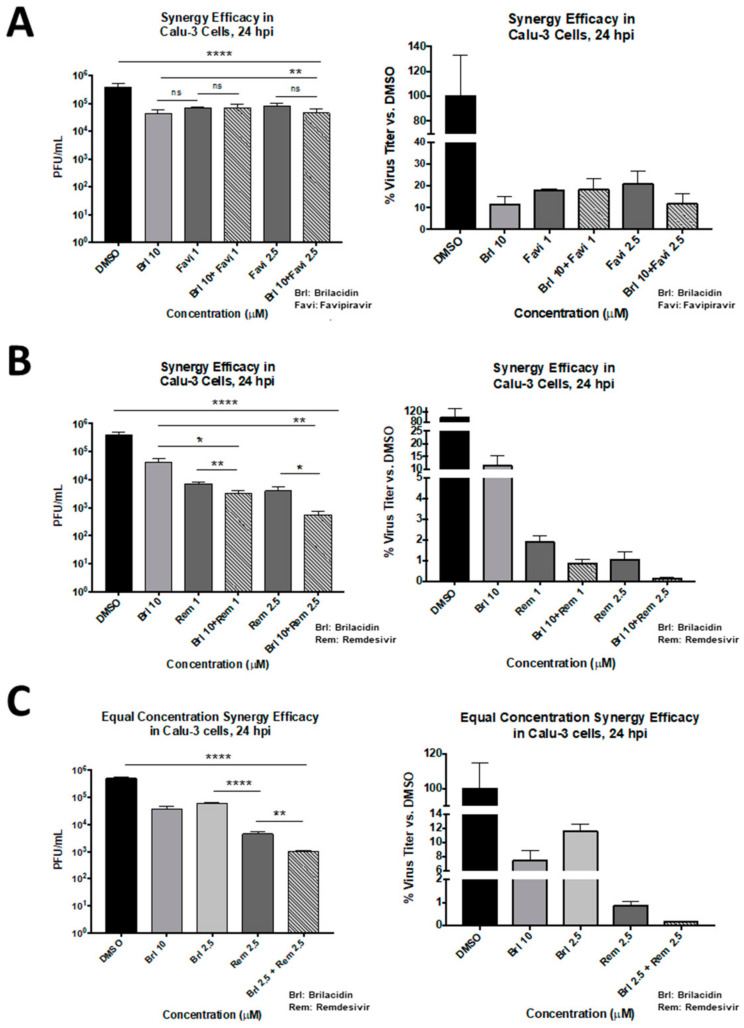
Efficacy of brilacidin as a combinatorial strategy as assessed in Calu-3 cells. Calu-3 cells were pretreated for 2 h with media alone or media containing brilacidin at 10 μM (for synergy treatments). (**A**) Cells were infected with SARS-CoV-2 at MOI 0.05 directly with brilacidin at 10 μM (for synergy treatments) or SARS-CoV-2 incubated in media alone (for favipiravir treatment alone). After 1 h, post-treatment with favipiravir alone or mixed with 10 μM brilacidin were added to cells at 1 or 2.5 μM concentrations. At 24 hpi, viral supernatants were evaluated by plaque assay as described in Materials and Methods. Calu-3 cells were pretreated for 2 h with media alone or media containing brilacidin at 10 or 2.5 μM (for synergy treatments). Cells were infected with SARS-CoV-2 at MOI 0.05 directly with brilacidin at 10 or 2.5 μM (for synergy treatments) or SARS-CoV-2 incubated in media alone (for remdesivir treatment alone). After 1 h, post-treatment with remdesivir alone, or mixed with 10 μM (**B**) or 2.5 μM (**C**) brilacidin, were added to cells at 1 or 2.5 μM concentrations. At 24 hpi, viral supernatants were evaluated by plaque assay as described in Materials and Methods. Graphs are representative of one independent experiment performed in technical triplicates (*n* = 3). Brl indicates brilacidin, Rem indicates remdesivir. Statistical analyses for synergy vs. individual control treatments were determined using Unpaired Two-Tailed Student’s *t* test. Significance against DMSO was determined as described in Materials and Methods. * *p* < 0.0332, ** *p* < 0.0021, **** *p* < 0.0001.

As brilacidin appears to act primarily by disrupting viral integrity and inhibiting viral entry, combining the drug with antiviral treatments that have a different mechanism of action may result in synergistic inhibition when administered in combination. The potential for brilacidin to exert a synergistic inhibition of SARS-CoV-2 when combined with current frontline COVID-19 antiviral treatments, namely, remdesivir and favipiravir, was assessed. Potential toxicity of combinations of remdesivir or favipiravir with brilacidin were initially assessed in the Calu-3 cell line at 24 h post-treatment. No apparent toxicity could be detected up to a 10 µM concentration of each of the drugs in the combination regimen. To evaluate the efficacy of combining remdesivir or favipiravir with brilacidin, the cells were pretreated with brilacidin for 2 h. The virus inoculum was also independently preincubated with brilacidin for 1 h, and then the treated inoculum was overlaid on cells and the infection allowed to proceed for 1 h in the presence of brilacidin. Postinfection, the inoculum was removed and media containing both brilacidin and remdesivir or favipiravir or each drug alone for efficacy comparison were added to the infected cells. Supernatants were obtained at 24 h postinfection and infectious titer was quantified by plaque assay. The data revealed that brilacidin and favipiravir independently exerted up to 90% and 80% inhibition, respectively, and the extent of inhibition did not increase over that exerted by brilacidin alone when the two drugs were used in combination (Figure 5A). In contrast, combination of brilacidin with remdesivir at 10 and 2.5 µM concentrations, respectively, reduced the viral infectious titer by >99%, thus providing a highly effective inhibition profile (Figure 5B) and achieving greater inhibition than with either compound alone. This synergistic inhibition continued to remain higher than 99% when the concentrations of both compounds were equal (2.5 µM each) (Figure 5C).

## 4. Discussion

The ongoing global COVID-19 pandemic powerfully reinforces the need for therapeutic strategies that can safely and effectively address virus- and host-based events elicited during SARS-CoV-2 infection.

In multiple studies, we have attempted to evaluate the capability of brilacidin to decrease viral load in the context of the SARS-CoV-2 infection. Our experiments in the Vero cell line model demonstrate brilacidin decreases viral load in a robust manner when the virus is preincubated with brilacidin (Figure 2D), suggesting brilacidin impacts virus integrity. Brilacidin’s ability to decrease viral load in an ACE2-positive cell line is demonstrated in Figure 3, Figure 4 and Figure 5, in which Calu-3 cells were used.

All experiments conducted in Vero and Calu-3 cell line models were supportive of an early inhibition exerted by brilacidin on SARS-CoV-2, indicating the drug’s impact on viral integrity. The idea that brilacidin directly interferes with the integrity of the virion is further supported by the observation that when drug treatment was limited to the virus alone (Figure 2E), with no treatment of host cells, a robust decrease of viral load was still observed in both the Washington strain and the Italian strain of SARS-CoV-2. This mechanism of inhibition may be akin to that achieved by neutralizing antibodies that may interact with specific exposed epitopes on the surface of virions. It remains to be determined if the impact of brilacidin on viral membranes is driven by specific viral membrane compositions.

While brilacidin’s mechanism of action appears primarily to be extracellular, it may also impact intracellular viral replication and is planned to be researched further. Supportive of this, an in silico quantum mechanical molecular screening study of 11,522 compounds identified brilacidin as a potential inhibitor of SARS-CoV-2 based on the potential of its physicochemical properties to interfere with the intracellular replication of SARS-CoV-2′s main protease (Mpro) [69].

The high CC50 (a measure of cytotoxicity) and low IC50 (a measure of potency) values observed for brilacidin in Calu-3 cells—yielding a Selectivity Index (SI) for brilacidin of 426 (CC50 = 241 μM/IC50 = 0.565 μM)—strongly support brilacidin’s treatment potential to achieve positive antiviral outcomes in humans. A vast majority of other drugs being evaluated as potential COVID-19 treatments, including repurposed drugs, have SIs that are much lower than that achieved by brilacidin [70], with most drugs failing to show anti-SARS-CoV-2 potency in the <1 μM range [71]. Of note, the IC50 (0.565 μM) and IC90 (2.63 μM) values for brilacidin observed in the Calu-3 cell line are well below clinically achievable concentrations based on pharmacokinetics observed in Phase 2 clinical trials with brilacidin for the treatment of Acute Bacterial Skin and Skin Structure Infections (ABSSSI). Applying the in vitro IC50 and IC90 parameter targets to in vivo human plasma concentration data, simulated dose regimens for brilacidin are similar to that already tested in clinical trials for ABSSSI and even exceed such targets, thereby further supporting the progression of brilacidin to clinical testing for treatment of COVID-19.

As of January 2021, FDA Investigational New Drug approval (with FDA Fast Track designation), and a similar regulatory approval from an overseas health authority, has been obtained for conduct of a multinational Phase 2 clinical trial of intravenously administered brilacidin in hospitalized patients with COVID-19. Brilacidin has been tested in numerous human trials (a total of eight) for other clinical indications, providing established safety and efficacy data on over 460 subjects.

A desirable outcome for any potential COVID-19 therapeutic will be its ability to synergize with existing COVID-19 treatments, particularly if the mechanisms of action of the synergistic treatments can impact more than one step of the viral lifecycle. Such combinations are more likely to elicit an additive response while also reducing the likelihood of viral resistance developing. Along these lines, we conducted experiments to evaluate the potential of brilacidin to work in conjunction with remdesivir and favipiravir (Figure 5), two frontline COVID-19 treatments, which proved supportive of synergistic inhibition between brilacidin and remdesivir. Remdesivir is a SARS-CoV-2 nucleotide analog RNA polymerase inhibitor that impacts the viral RNA synthesis step of the infectious process. By that mechanism, remdesivir may help decrease progeny viral genomes in infected cells but will not be conducive to inhibiting progressive infection of naïve cells once the progeny virions have been released from infected cells.

By combining remdesivir with brilacidin, a two-pronged strategy of inhibiting viral entry and viral RNA synthesis might be successfully leveraged to most effectively control progression of SARS-CoV-2 infection. The opportunity that combination treatments with brilacidin could potentially offer in treating COVID-19 requires further exploration, and in vivo animal studies are in planning stages.

Clearly, an effective COVID-19 therapeutic (or therapeutics in combination) ideally would control both viral load and the corresponding inflammatory damage due to SARS-CoV-2 [72], and mitigate bacterial coinfections. With its HDP mimetic properties—antiviral, immuno/anti-inflammatory, and antibacterial—brilacidin may be able to address the different disease parameters of COVID-19 within the one therapeutic treatment. The results of the planned Phase 2 clinical trial, with intravenous treatment of COVID-19 in addition to standard of care, are highly anticipated.

In this manuscript, we demonstrate brilacidin exhibits robust inhibition of SARS-CoV-2 in Vero cells and Calu-3 cells, and in two strains of the virus. Likely to function as a viral entry inhibitor [73,74,75], the proposed mechanism of action for brilacidin includes affecting the integrity of the viral membrane and interfering with viral entry. Brilacidin also exhibited an excellent synergistic inhibitory profile against SARS-CoV-2 in combination with remdesivir. Destabilizing viral integrity is a desirable antiviral property, especially in relation to pan-coronavirus agents, as the viral membrane is highly conserved and similar in construct across different coronavirus strains. Further research will be conducted in the context of other lethal coronaviruses (MERS-CoV, SARS-CoV) toward assessing the potential of brilacidin as a broad-spectrum inhibitor of coronaviruses.

## Data Availability

All new data generated for this publication have been included in the current manuscript.
